# Aberrant olfactory network functional connectivity in people with olfactory dysfunction following COVID-19 infection: an exploratory, observational study

**DOI:** 10.1016/j.eclinm.2023.101883

**Published:** 2023-03-02

**Authors:** Jed Wingrove, Janine Makaronidis, Ferran Prados, Baris Kanber, Marios C. Yiannakas, Cormac Magee, Gloria Castellazzi, Louis Grandjean, Xavier Golay, Carmen Tur, Olga Ciccarelli, Egidio D'Angelo, Claudia A.M. Gandini Wheeler-Kingshott, Rachel L. Batterham

**Affiliations:** a Centre for Obesity Research, Department of Medicine, University College London, London, UK; b National Institute for Health and Care Research, Biomedical Research Centre at UCLH and UCL, London, UK; c NMR Research Unit, Queen Square MS Centre, Department of Neuroinflammation, UCL Queen Square Institute of Neurology, University College London, London, UK; d Centre for Medical Image Computing, Department of Medical Physics and Biomedical Engineering, University College London, London, UK; e Brain Connectivity Research Centre, IRCCS Mondino Foundation, Pavia, Italy; f Department of Infection, Immunity & Inflammation, UCL Great Ormond Street Institute of Child Health, University College London, London, UK; g Department of Brain Repair and Rehabilitation, UCL Queen Square Institute of Neurology, University College London, London, UK; h Department of Brain and Behavioural Sciences, University of Pavia, Pavia, Italy

**Keywords:** COVID-19 anosmia, Olfactory impairments, Functional neuroimaging, Olfactory networks, Resting state connectivity, Cerebral blood flow

## Abstract

**Background:**

Olfactory impairments and anosmia from COVID-19 infection typically resolve within 2–4 weeks, although in some cases, symptoms persist longer. COVID-19-related anosmia is associated with olfactory bulb atrophy, however, the impact on cortical structures is relatively unknown, particularly in those with long-term symptoms.

**Methods:**

In this exploratory, observational study, we studied individuals who experienced COVID-19-related anosmia, with or without recovered sense of smell, and compared against individuals with no prior COVID-19 infection (confirmed by antibody testing, all vaccine naïve). MRI Imaging was carried out between the 15th July and 17th November 2020 at the Queen Square House Clinical Scanning Facility, UCL, United Kingdom. Using functional magnetic resonance imaging (fMRI) and structural imaging, we assessed differences in functional connectivity (FC) between olfactory regions, whole brain grey matter (GM) cerebral blood flow (CBF) and GM density.

**Findings:**

Individuals with anosmia showed increased FC between the left orbitofrontal cortex (OFC), visual association cortex and cerebellum and FC reductions between the right OFC and dorsal anterior cingulate cortex compared to those with no prior COVID-19 infection (*p* < 0.05, from whole brain statistical parametric map analysis). Individuals with anosmia also showed greater CBF in the left insula, hippocampus and ventral posterior cingulate when compared to those with resolved anosmia (*p* < 0.05, from whole brain statistical parametric map analysis).

**Interpretation:**

This work describes, for the first time to our knowledge, functional differences within olfactory areas and regions involved in sensory processing and cognitive functioning. This work identifies key areas for further research and potential target sites for therapeutic strategies.

**Funding:**

This study was funded by the 10.13039/501100000272National Institute for Health and Care Research and supported by the Queen Square Scanner business case.

**Research in context**

**Evidence before this study**

We searched PubMed for original research articles using a combination of terms for “COVID-19”, “Anosmia”, “olfactory dysfunction”, “neuroimaging”, “brain function” from study commencement (July 2020) and until the production of the work (July 2022). We found a number of case studies looking at the impact of acute COVID-19 related anosmia on brain structure. These data found injury and atrophy to the primary odour sensing regions, the olfactory bulbs. In addition, a large-scale UK Biobank study looking at COVID-19 more generally identified structural changes in cortical regions involved in odour processing. There is limited data on the impact of long-COVID anosmia on brain function.

**Added value of this study**

These data show that there are functional connectivity changes in secondary odour processing regions as well as regions involved in cognitive processing as a result of long-COVID anosmia compared to healthy controls (not exposed to COVID-19). In addition, we identified cerebral perfusion differences within olfactory regions between individuals who regain their sense of smell and those still suffering with long-COVID anosmia.

**Implications of all the available evidence**

Our data highlights the impact of COVID-19 anosmia on brain functional connectivity and cerebral perfusion. These data open up potential avenues for treatments and ways to measure olfactory training success for those still suffering with long-COVID.

## Introduction

Within a few months of the onset of the COVID-19 pandemic, impaired olfactory function, either partial smell loss (hyposmia) or complete loss (anosmia) was recognised as an acute symptom of COVID-19 infection. Indeed, impaired olfactory function was found to be one of the best predictors of COVID-19 in individuals with recent respiratory symptoms.^1^, ^2^, ^3^, ^4^ In most individuals, their sense of smell recovers within 7–14 days.^5^ However, in others, olfactory dysfunction persists for several weeks or in some cases, longer, impacting upon their quality of life. Persistent symptoms following COVID-19 infection beyond 12 weeks is defined as ‘long COVID’ and persistent anosmia can be a manifestation of long COVID.^6^

Structural neuroimaging case studies looking at acute COVID-19 associated smell loss have shown atrophy and injury to the primary odour sensing region, the olfactory bulbs.^7^ In addition to the olfactory bulbs, there are secondary regions within the cortex that are involved in odour processing, rather than sensing, such as the piriform cortex (Pir) and orbitofrontal cortex (OFC)^8^ and it is unknown whether these regions are affected by pathological changes in cases of persistent anosmia. Sensory loss in general is known to manifest in neuronal and morphological changes within the brain, predominantly explained by the loss of sensory input.^9^ Magnetic resonance imaging (MRI) based research has offered insights into some of these morphological and functional changes seen following anosmia, not related to COVID-19, within the cortical olfactory regions.^10^

Task-based functional MRI (fMRI) research has shown impaired regional activation in response to olfactory stimuli in patients with olfactory loss (not COVID-19 related) compared to healthy controls,^11^ notably in the OFC. fMRI data acquired during rest (i.e. when not engaged in a task) is known as resting state fMRI (rs-fMRI) and has identified that olfaction is processed as part of a network, known as the olfactory network,^12^ which incorporates both the Pir, OFC and insula cortex.^8^ rs-fMRI relies on the blood oxygenation level dependent (BOLD) contrast and is an ideal method to investigate local fluctuations of signals linked to basal brain activity in relevant cortical areas. Moreover, from rs-fMRI it is possible to perform seed-based analysis and assess functional connectivity (FC) between a pre-defined seed region and the rest of the brain. This permits, hypothesis-driven analysis or the study of so-called resting state networks, ensembles of regions that have a consistent pattern of signal fluctuations and that emerge when performing signal fluctuation analysis of the signal at whole brain level. There are a small number of well-studied and defined resting state networks, each associated with a particular set of functions, e.g. sensory, cognitive or associative functioning.

High-resolution anatomical images, such as those acquired using MRI sequences (e.g. a 3D T1-weighted gradient echo sequence), allow estimation of cortical structural changes and can be used to assess local changes in tissue density. Thus, reflecting either brain swelling or shrinkage that have been associated with inflammation and atrophy, respectively. To date, the largest study looking at the impact of COVID-19 on the brain, comes from the analysis of structural MRI data from the UK Biobank.^13^ In this work, 351 individuals who tested positive for COVID-19 and who also had prior baseline imaging data, showed decrease in cortical thickness in the left lateral OFC following COVID-19 infection.^13^ What is important to note here is that information regarding anosmia/hyposmia symptoms were not collected from these participants, which makes it difficult to make specific inferences from this data and therefore requires research that utilises better characterised participant cohorts.

To this end, our previous work, undertaken in London, UK, during the first-wave of the COVID-19 pandemic was one of the first community cohort studies to describe the association between COVID-19 infection with both loss of smell and taste.^1^ People recruited to the study were not hospitalised and were vaccine naïve due to the study being conducted prior to vaccine development. From this cohort a number of individuals who still had impaired olfactory function 4–6 weeks after initial COVID-19 infection were identified.^14^ *Makaronidis* et al., described an effect of age on smell resolution, with younger individuals more likely to fully resolve their smell and older individuals less likely.^14^ However, the mechanisms underlying ongoing impaired olfaction and in particular changes in brain function and structure were largely unknown. Therefore, in the summer of 2020, an MRI project^15^ was initiated to explore two main questions in non-hospitalised people who suffered from COVID-19 infection and acute anosmia: firstly, what is the impact of COVID-19 associated olfactory loss on brain function and structure compared to people who did not experience infection? and secondly, can we identify differences between those individuals who recover their smell and those who do not? To address these questions, we utilised multi-modal MR imaging techniques to assess how COVID-19 infection and related long-COVID anosmia impacts on resting state FC, cerebral blood perfusion and GM density.

Here we present the results from the rs-fMRI seed-based analysis of this study, focusing on COVID-19 associated anosmia and explore the contribution of atrophy and blood perfusion to understand possible mechanisms of dysfunction.

## Methods

### Study design and participants

A total of fifty-seven individuals were recruited for this exploratory, observational study and stratified into five groups based on COVID-19 antibody (IgG/IgM) status and also, if characterised, chemosensory status.

Individuals with COVID-19 infection associated chemosensory impairments were recruited from the *Makaronidis* et al., study described above.^14^ These individuals were stratified into a group whose anosmia symptoms remained for greater than 4 weeks following initial symptom and infection onset (referred herein as the CoV+*Anos-Long* group), an age-matched group whose sense of smell had fully recovered (CoV+*Anos-Recov*) and a group of significantly younger individuals (<30 years old, all group demographic data are reported in Table 1) also with fully resolved sense of smell (*CoV+Anos-Young*). We recruited participants from the community who had mild COVID-19, in that they did not require hospitalisation.

**Table 1:** Demographic data for each group.

Group	Age (years)	Female (Male)	UPSIT score
Cntrl (n = 18)	38.89 ± 11.39	9 (9)	–
CoV (n = 11)	37.02 ± 9.08	8 (3)	–
CoV+Anos-Long (n = 8)	52.25 ± 12.17	7 (1)	15.63 ± 5.85
CoV+Anos-Recov (n = 10)	50.79 ± 8.91	9 (1)	34.10 ± 1.85
CoV+Anos-Young (n = 10)	27.73 ± 1.87	6 (4)	34.23 ± 2.00

University of Pennsylvania Smell Identification Test (UPSIT) scores were recorded at the time of scanning but were only recorded for the three CoV-Anos groups. Cntrl: Healthy control, CoV: Previous COVID-19 infection, CoV+Anos-Long: long COVID-19 Anosmia, CoV+Anos-Recov: Recovered COVID-19 Anosmia (age-matched to CoV+Anos-Long) and CoV+Anos-Young: Younger in age Recovered COVID-19 Anosmia group.

Chemosensory perception was assessed using the University of Pennsylvania Smell Identification Test (UPSIT, MediSense, www.smelltest.eu). The UPSIT test is a 40-item ‘scratch and sniff’ test, which has been validated for self-administration.^16^ The UPSIT generates a score out of 40, which following adjustment for age and sex, classifies olfactory function into normosmia, mild microsmia, moderate microsmia, severe microsmia and total anosmia.^16^ At the time of scanning the *CoV+Anos-Long* group had an UPSIT score that described total anosmia or severe microsmia. The two *CoV+Anos* resolved groups had individual UPSIT scores indicating normosmia at the time of scanning. MRI Imaging was carried out between the 15th July and 17th November 2020 at the Queen Square House Clinical Scanning Facility, UCL, United Kingdom. Onset of smell loss was between the 7th March and 20th April 2020, according to the recruitment of *Makaronidis* et al.^14^ UPSIT testing was performed at the time of scanning for these anosmia individuals and the mean duration from symptom onset to MRI scanning were 168 ± 43 days (mean ± standard deviation (SD)).

To make comparisons with healthy individuals we recruited individuals who presented with a negative COVID-19 antibody test on the day of scanning (*Cntrl*). Moreover, these healthy individuals did not undergo a clinical assessment of olfactory function. The fifth group were formed of individuals who presented with a positive SARS-CoV antibody test, but who had no clinical assessment of olfactory function or other symptoms (*CoV*). This group were age-matched to the *Cntrl* group. The *Cntrl* and *CoV* cohorts were recruited from self-referrals or from word of mouth.

Exclusion criteria for this imaging study consisted of any MR scanner contraindications (aneurysm clips, pacemakers, metallic implants, pregnancy), previous diagnosis of a neurological condition likely to affect imaging measures. Table 1 presents demographic data for this cohort. Written informed consent was provided by all individuals prior to data collection.

### Image acquisition

MRI data were acquired using a 3T Philips Ingenia CX system (Philips Healthcare, Best, The Netherlands) with a 32-channel head coil. For all individuals a comprehensive structural and functional protocol was acquired,^15^ including a clinical 3D fluid attenuated inversion recovery (FLAIR) sequence to assess for brain abnormalities, which underwent radiological review by an experienced neuroradiologist. For the purpose of this study, we used data acquired for structural imaging, rs-fMRI and ASL. Details are given here below:

1. Structural imaging—high resolution (1 mm^3^ isotropic) 3D T1 weighted images were acquired for brain volumetric measurements as well as for co-registration and normalisation of functional imaging data to the Montreal Neurological Institute (MNI) standardised space. A magnetisation prepared 3D Turbo Field Echo sequence was used with the following parameters: slice thickness (Δ*z*) = 1 mm, number of slices = 176, TR = 28 ms, Echo time (TE) = 7 ms, flip angle (FA) = 24°, matrix size (DM) = 256 × 256 and field of view (FOV) = 256 × 256mm^2^, acquired in the sagittal plane. Acquisition time: 4:06 min.
2. rs-fMRI—resting-state T2∗ weighted images sensitive to BOLD signal fluctuations were acquired using a 2D echo planar imaging (EPI) sequence with the following parameters: Δ*z* = 3 mm, slices = 43, slice gap = 0.5 mm, TR = 4000 ms, TE = 25 ms, FA = 90° and reconstructed voxel size = 2.88 × 2.88 × 3 mm^3^. Slices were acquired in an interleaved order with a total acquisition time = 6 m 40 s with 100 repeated dynamics. Individuals were asked to stay still and keep their eyes open for the duration of the scan.
3. Perfusion imaging at rest—whole brain cerebral perfusion images were collected according to the ‘White Paper’ recommendations^17^ at rest, using a 3D pseudo-continuous arterial spin labelling (pCASL) sequence acquired with a gradient and spin echo (GRASE) multi-shot readout and the following parameters: label duration = 1800 ms, post-labelling delay = 2000 ms, TE = 12 ms, TR = 4266 ms, total acquisition time = 7 m 15 s. A 4-pulse background-suppression scheme was also applied to null static tissue signal. As part of the pCASL sequence a 3D proton density (PD) image using identical readout parameters is acquired for cerebral blood flow quantification purposes and was also used to aid co-registration between anatomical T1 images. CBF maps were computed at the voxel level from perfusion weighted (PW) and PD images using the single compartment model as provided by the Philips scanner software.

### Image pre-processing

A combination of imaging software was used for both the pre-processing and analysis of imaging data, namely Statistical Parametric Mapping Software (SPM-12, Functional Imaging Laboratory (FIL), The Wellcome Trust Centre for NeuroImaging, Queen Square Institute of Neurology, University College London (UCL), UK, http://www.fil.ion.ucl.ac.uk/spm/software/spm12/), Advanced Functional NeuroImaging Software (AFNI, https://afni.nimh.nih.gov/) and NiftySeg Software (Centre for Medical Image Computing, UCL, UK).

### Structural pre-processing

3D T1 weighted images were segmented into tissue based probabilistic images (Grey matter (GM), white matter (WM) and cerebrospinal fluid (CSF)) using *NiftySeg* software and normalised to MNI atlas space. Once normalised to this atlas space GM probabilistic images were smoothed using an 8 × 8 × 8 mm^3^ full width at half maximum (FWHM) gaussian kernel.

### BOLD pre-processing

Pre-processing of BOLD timeseries data was performed in accordance with the Enhancing Neuro Imaging Genetics through Meta Analysis (ENIGMA) proposed pipeline.^18^ Firstly, EPI data were denoised using a custom made Marchenko-Pastur principal component analysis (MP-PCA) method to identify and subsequently remove principal components which originate from thermal noise. Denoised timeseries were despiked, before performing slice timing and head motion correction.^19^ Average signal timeseries were extracted from WM and lateral ventricle (CSF) masks. These time courses were later used for nuisance regression of confounding signals. Co-registration of EPI to T1 weighted images was conducted using the NiftyReg software while normalisation of rs-fMRI data to MNI space was performed by applying the saved affine transformation parameters from the structural analysis; finally images were smoothed (AFNI) with a 6 mm^3^ FWHM Gaussian Kernel. 6 motion parameters (3 rotation and 3 translation), their 6 temporal derivatives and the signal time courses from the WM and CSF regions were regressed from the BOLD timeseries, for a total of 14 regressors (AFNI).

### ASL pre-processing

For the pre-processing of perfusion data, we applied a two-step approach to align the CBF maps to MNI space. Firstly, the PD images, which are in perfect alignment with CBF maps and boast higher tissue contrast, were co-registered to the high resolution 3D T1 images using *NiftyReg.* The transformation parameters were saved and subsequently applied to the CBF maps. Secondly, these CBF map, now co-registered to T1 space, were normalised to MNI space by applying the previously saved affine registration parameters (T1 to MNI). CBF maps in standardised space were smoothed using a FWHM Gaussian kernel of 8 mm^3^.

### Voxel based morphometry

Voxel-based morphometry analysis was performed to identify volumetric GM differences between groups. Group differences were assessed through a second level mass univariate analysis using SPM-12. GM probability maps for each subject were entered into a one-way ANOVA with age and sex as nuisance covariates. To account for global differences in tissue volume Total Intracranial Volume (TIV), which was calculated from segmentation data, was added as an additional covariate for each subject to the model. For comparisons across groups, we looked at all comparisons by creating linear contrasts. All pairwise group contrasts were tested and parametric contrast t-maps were created using a family wise error (FWE) cluster forming threshold *p* < 0.05. Significant clusters that survived this threshold were assessed at *p* < 0.05 using FWE.

### Connectivity analysis

Two types of FC analysis were conducted: a seed-based analysis which had a greater focus on regions perhaps affected by anosmia and a dual regression analysis which interrogated large, well characterised connectivity networks.

**Fig. 1:**
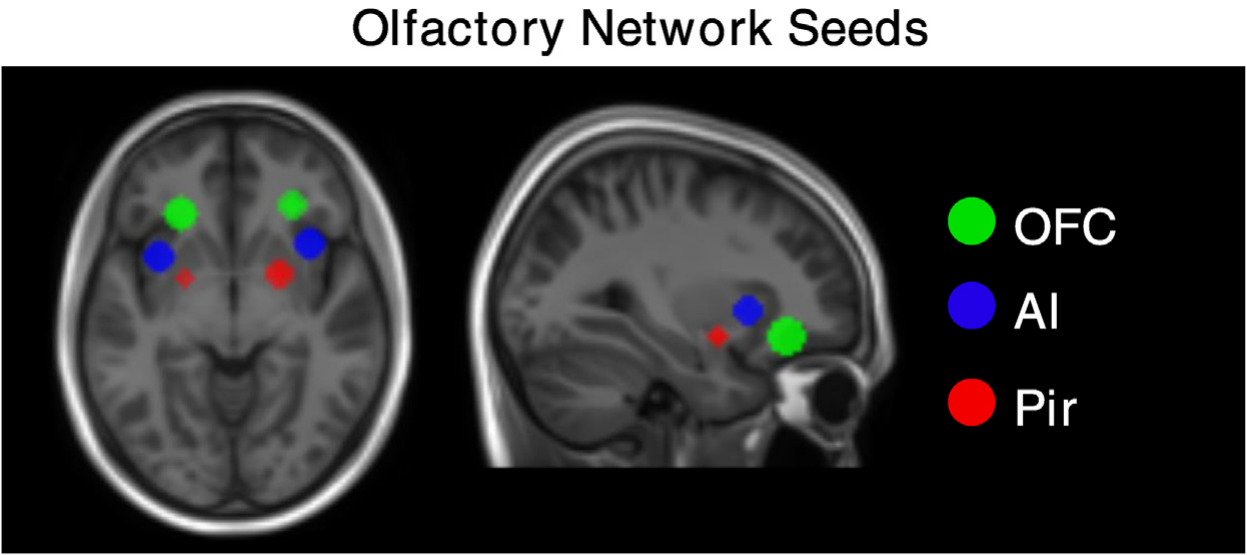
**Anatomical image displaying the locations of the bilateral seeds used for the connectivity analysis, comprising the olfactory network.** In green the orbitofrontal cortex (OFC), in blue the anterior insula (AI) and in red the Piriform Cortex (Pir). Clusters were defined based on co-ordinates from Tobia et al. Each seed is 9 mm in diameter.

For the seed-based analysis we referred to *Peter* et al.^20^ who previously investigated resting state olfactory networks comprising 6 anatomical nodes; bilateral OFC, anterior insula (AI) and piriform cortex (Pir). 9 mm diameter seeds for these regions were created using the co-ordinates published in *Tobia* et al.^12^ (Fig. 1). Seed time courses were extracted and regressed against processed EPI data for each individual. Using Fisher's r-to-z-transformation these correlation maps were then transformed to produce connectivity z-maps for each individual and for each individual seed.

Dual regression analysis^21^ was performed using template resting state networks from the ENIGMA pipeline. These networks were defined based on the probabilistic regions of interest (ROIs) from 20-component analysis of the BrainMap activation database and resting fMRI dataset. The resting state template networks corresponded to the auditory network (AN), default mode network (DMN), fronto-parietal network (FPN), sensorimotor network (SMN), visual network (VN), executive control network (ECN), salience network (SN), and attention network (AttN).

First, for each individual, the template derived set of spatial maps was regressed (as spatial regressor in a multiple regression) into the single individual's 4D space-time dataset, one per group-level spatial map. Next, those time series were regressed as temporal regressors, again in a multiple regression, into the same 4D dataset, resulting in a set of individual-specific spatial maps, one per group-level spatial map. Mean time series were extracted from each network across all individuals and, for each individual regressed (using multiple regression) into each individual's processed EPI timeseries. This resulted in a set of individual-specific time series, for each network. Next, those time series were regressed as temporal regressors, again in a multiple regression, into the same 4D dataset, resulting in a set of individual-specific spatial maps, which were transformed to z-maps using Fisher's r-to-z transformation. These connectivity z-maps were used for group level analysis.

Group connectivity differences were assessed through a second level mass univariate analysis using SPM software. For each seed z-map a one-way ANOVA with age and sex as covariates was constructed, and all pairwise group contrasts were tested. Parametric contrast t-maps were created with a cluster forming threshold of *p* < 0.001. Significant clusters were assessed at *p* < 0.05 family wise error (FWE) threshold. Similarly, one-way ANOVA models with age and sex as covariates were constructed for each resting state network and assessed using the same statistical thresholds as defined above.

To aid interpretation, significant clusters, were saved and used as masks to extract mean signal from connectivity z-maps for all individuals. No statistical tests were performed on these extracted values. In addition, the Yale BioImage Suite Medical Image Analysis Software (https://bioimagesuiteweb.github.io/webapp/mni2tal.html) was used for anatomical referencing cerebrum structures and the cerebellar Atlas within the Functional Software Library (FMRIB software library, Oxford, UK) was used for referencing cerebellar structures.

### CBF analysis

Cerebral perfusion differences across groups were assessed through a second level mass univariate analysis using SPM. Processed CBF maps were entered into a voxel-wise one-way ANOVA with age and sex as covariates and all pairwise group contrasts were tested. In addition, mean GM CBF values were calculated from a GM mask and added to this model as covariates to account for inter individual differences in global perfusion. The GM mask was calculated from a template T1-based probabilistic map of GM distribution by thresholding all voxels with a probability >0.20. Analysis was constrained to those voxels within the GM by using this GM mask as an implicit mask for our whole brain analysis. Similar to the connectivity analysis above, parametric contrast t-maps were created for each contrast with a cluster forming threshold of *p* < 0.001. Significant clusters were assessed at *p* < 0.05 family wise error (FWE) threshold.

In addition, using the GM mask described above mean GM values were extracted and entered into a one-way ANOVA model to assess whether any global cerebral blood differences existed between the groups.

### Statistical analysis

Whole brain statistical analyses were carried out using SPM-12 software. To help visualise significant differences across groups, we used significant clusters as masks to extract average values from z-maps and CBF maps which have been plotted alongside statistical parametric maps using R statistical analysis software (Rstudio—Version 1.1.453, Boston, MA, http://www.rstudio.org/). Descriptive parametric statistics were used to describe the results and reported as mean ± SD unless stated otherwise.

### Study approval

This study received ethical approval by the Queen Square Research Ethics Committee (IRAS Project ID 282668, ClinicalTrials.gov: NCT04377815) and was conducted in line with the declaration of Helsinki and the standards of Good Clinical Practice. The ‘Development of Novel MRI Techniques for Neurological Applications’ study, which was responsible for recruiting and scanning the healthy control and CoV participants, received ethics approval from the London Harrow Research Ethics Committee (05/Q0502/101) and was conducted in line with the declaration of Helsinki and the standards of Good Clinical Practice.

### Role of the funding source

The funders had no role in the study design, delivery or analysis of the observational study results or writing the report. All authors are responsible for the decision to submit the manuscript and all authors had access to the data and have read and approved the final version of the manuscript.

## Results

Individual demographics are reported in Table 1. To clarify our stratification, of the 57 individuals in this study 28 individuals were recruited from the *Makaronidis* et al., study cohort and when discussed collectively, will be known as the CoV-Anos groups.^1^^,^^14^ All 28 individuals had a previous positive antibody test and had suffered with COVID-19 associated anosmia. 20 of these individuals recovered their sense of smell prior to scanning, whereas 8 did not. The 8 who did not regain their sense of smell will be identified as the *CoV+Anos-Long* group (n = 8). The 20 individuals who *recovered* their sense of smell have been divided into two groups; one which is age matched to the *Cov+Anos-Long* group and is known herein as the *CoV+Anos-Recov group (n = 10)* and the other with a lower mean age, known herein as the *CoV+Anos-Young* group (n = 10). Furthermore, healthy control individuals (*Cntrl, n = 18*) with negative antibody tests at the time of screening were included as well as individuals with a positive antibody test, but with no formal symptom assessment were also included, identified as the *CoV* group (n = 11). These two groups were matched for age on a case-by-case basis and did not differ in age (see Table 1). ANOVA analysis revealed significant group effects with respect to age (see supplementary material, Fig S1).

Overall, we found functional BOLD and CBF differences as well as structural differences between groups. Specific results of the various tests and group differences are reported here below. We report the functional BOLD and CBF results followed by the structural findings.

### Whole brain resting state functional connectivity analysis—seed-based analysis

All FC results are presented in Table 2. We found both increases and decreases in FC between the *CoV+Anos-Long group* and the *Cntrl group,* and are reported in this order below.

**Table 2:** Functional connectivity results.

Seed/Network	Contrast	Region	MNI co-ordinates			Cluster size (voxels)	*p* value
			x	y	z		
R-OFC	CoV-Anos-Long > Cntrl	Right visual association cortex and fusiform gyrus	50 46	−70 −56	12 −4	191	<0.05
R-OFC	CoV-Anos-Long > CoV	Right visual association cortex and fusiform gyrus	48 50	−66 −70	0 10	242	<0.05
L-AI	CoV-Anos-Long > Cntrl	Left Crus I cerebellum	−54 −18	−48 −78	−32 −22	191	<0.05 <0.05
L-OFC	CoV-Anos-Long < Cntrl	Left dorsal ACC	−8	38	−2	242	<0.01
EC network	CoV-Anos-Long < Cntrl	dorsal ACC	−4	42	2	180	<0.05

OFC: orbitofrontal cortex, AI: Anterior Insula, ACC: Anterior Cingulate Cortex, EC: Executive Control, Cntrl: Healthy controls, CoV: Previous COVID-19 infection, CoV+Anos-Long: Long COVID-19 Anosmia, CoV+Anos-Recov: Recovered COVID-19 Anosmia (age-matched to CoV+Anos-Long) and CoV+Anos-Young: Young and Recovered COVIID-19 Anosmia group.

### Long COVID-19 anosmia—increased functional connectivity

**Fig. 2:**
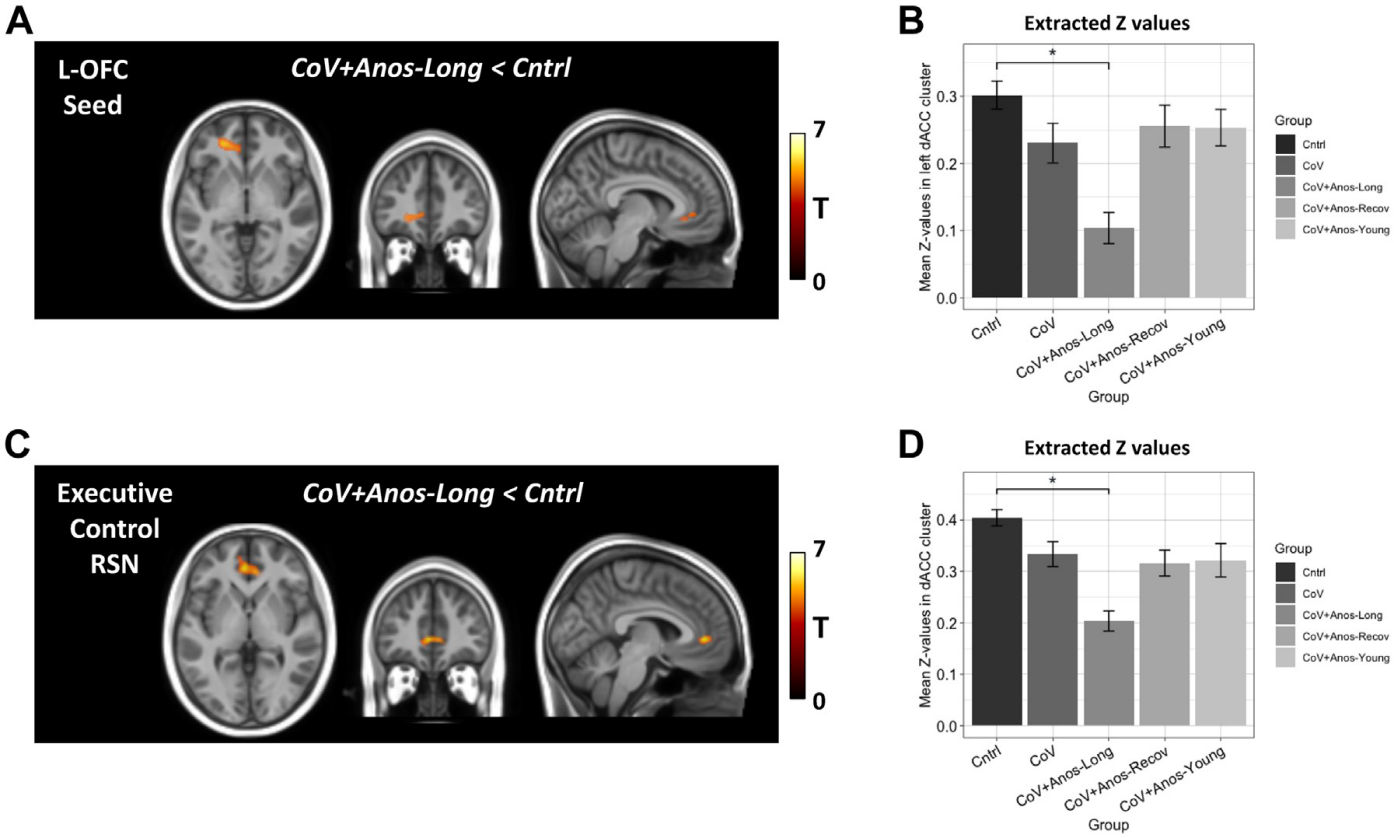
**A) In hot colours, significant clusters overlaid on the MNI template showing increased functional connectivity between the right OFC seed and the right visual association cortex/fusiform gyrus, in the *CoV+Anos-Long* group compared to the *Cntrl* group. B) Mean Z values extracted from the right fusiform cluster for each group. C) Significant clusters overlaid onto an MNI template showing increased connectivity between the left anterior insula seed and the left Crus I region of the cerebellum, in the *CoV+Anos-Long* group compared to the *Cntrl* group. D) Mean Z values extracted from the left Crus I cluster for each group.** ∗ denotes the significant group comparisons for whole brain analysis, statistics was not performed on extracted values. Both Statistical Parametric Maps were obtained using a cluster significance threshold set to *p* < 0.05.

Group level analysis of the right OFC (R-OFC) seed maps yielded a significant cluster (*p = 0.04, T = 4.27*) in the right occipital cortex, predominantly spanning the right visual association cortex but also the fusiform gyrus, which was increased in the *CoV+Anos-Long* group compared to the *Cntrl* group (Fig. 2A). In addition, the *CoV+Anos-Long* group displayed increased FC between the R-OFC and the same occipital/fusiform region compared to the *CoV* group.

When looking at the anterior insula (AI) the *CoV+Anos-Long* individuals compared to *Cntrl*, displayed an increase in FC between the left AI (L-AI) seed and two significant clusters located in the cerebellum, both corresponding to the left Crus I region (Fig. 2C).

### Long COVID-19 anosmia—decreased functional connectivity

**Fig. 3:**
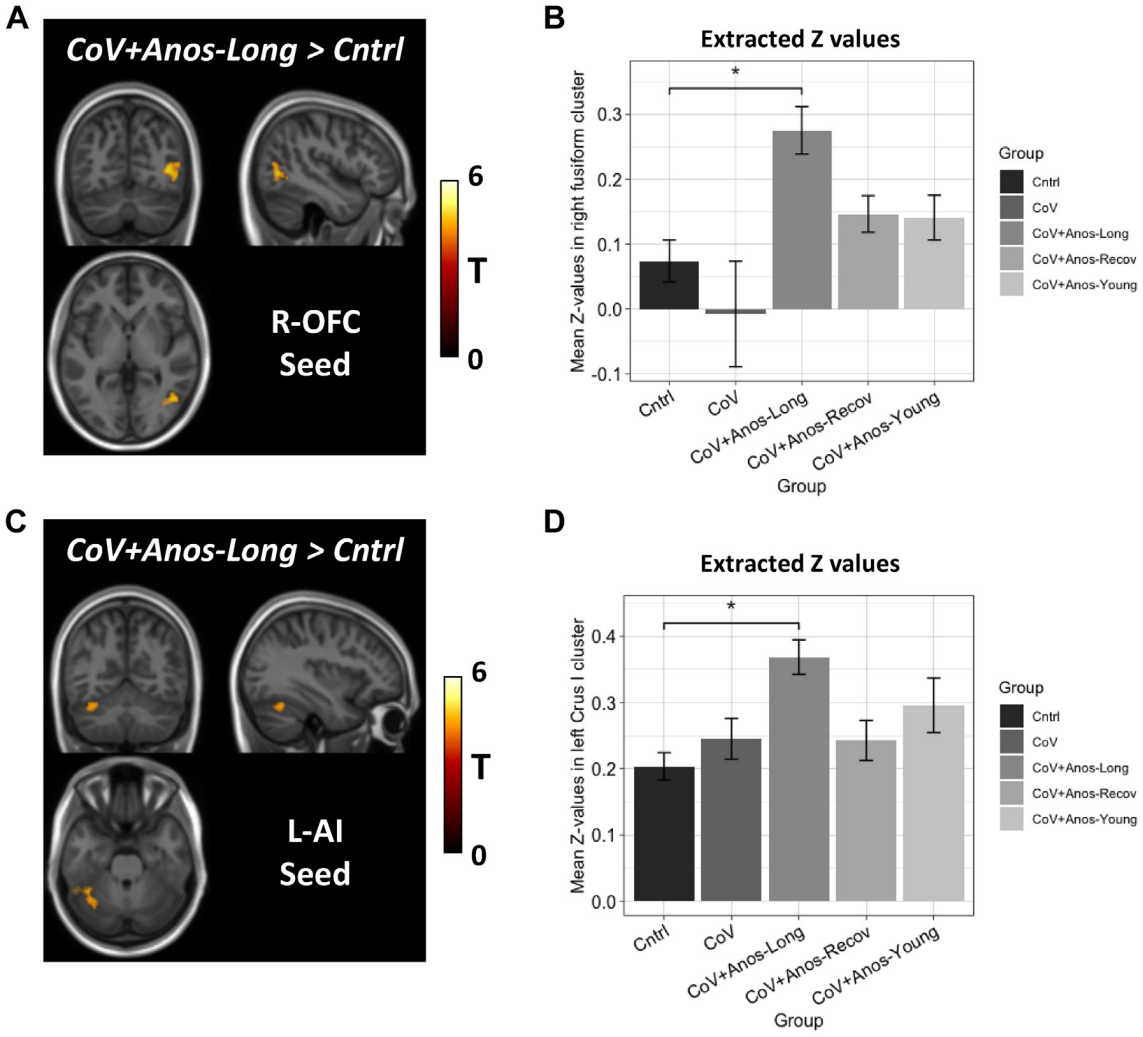
**A) In hot colours, significant clusters overlaid on the MNI template showing reduced functional connectivity between the left OFC seed and the left dorsal anterior cingulate cortex, in the *CoV+Anos-Long* group compared to the *Cntrl* group. B) Mean Z values extracted from left dorsal anterior cingulate cluster for each group. C) Significant clusters overlaid onto an MNI template showing reduced connectivity in the executive control network with one of the main nodes, the dorsal anterior cingulate cortex, in the *CoV+Anos-Long* group compared to the *Cntrl* group. D) Mean Z values extracted from the anterior cingulate cluster for each group.** ∗ denotes the significant group comparisons for whole brain analysis, statistics was not performed on extracted values. Both Statistical Parametric Maps were obtained using a cluster significance threshold set to *p* < 0.05.

When comparing *CoV+Anos-Long* with the *Cntrl* group, the left OFC (L-OFC) seed analysis showed a significant (*p* = 0.01, T = 4.79) reduction in FC between the L-OFC seed region and a cluster within the left Dorsal Anterior Cingulate Cortex (dACC) in the *CoV+Anos-Long* compared to *Cntrl* (Fig. 3A).

### Resting state network analysis

When comparing resting state network FC between groups only one significant group difference was identified within the executive control network. Our analysis showed that there was a significant reduction in FC within this network in a cluster (180 voxels) found in the region of the dACC in the *CoV+Anos-Long* group compared to the *Cntrl* group (*p* < 0.05, *T* = 5.29, Fig. 3C). No other differences were found for this network for any of the other comparisons.

### Whole brain cerebral blood flow analysis

Prior to conducting whole brain analysis, group differences in GM CBF were assessed. There were no significant main group effects identified (*F* = 0.85).

Whole brain CBF analysis revealed group differences between the *CoV+Anos-Long* group and the other two *CoV+Anos* groups. In both cases the *CoV+Anos-Long* group displayed increased regional CBF in comparison to the *CoV+Anos-Recov* and *CoV+Anos-Young* groups.

**Fig. 4:**
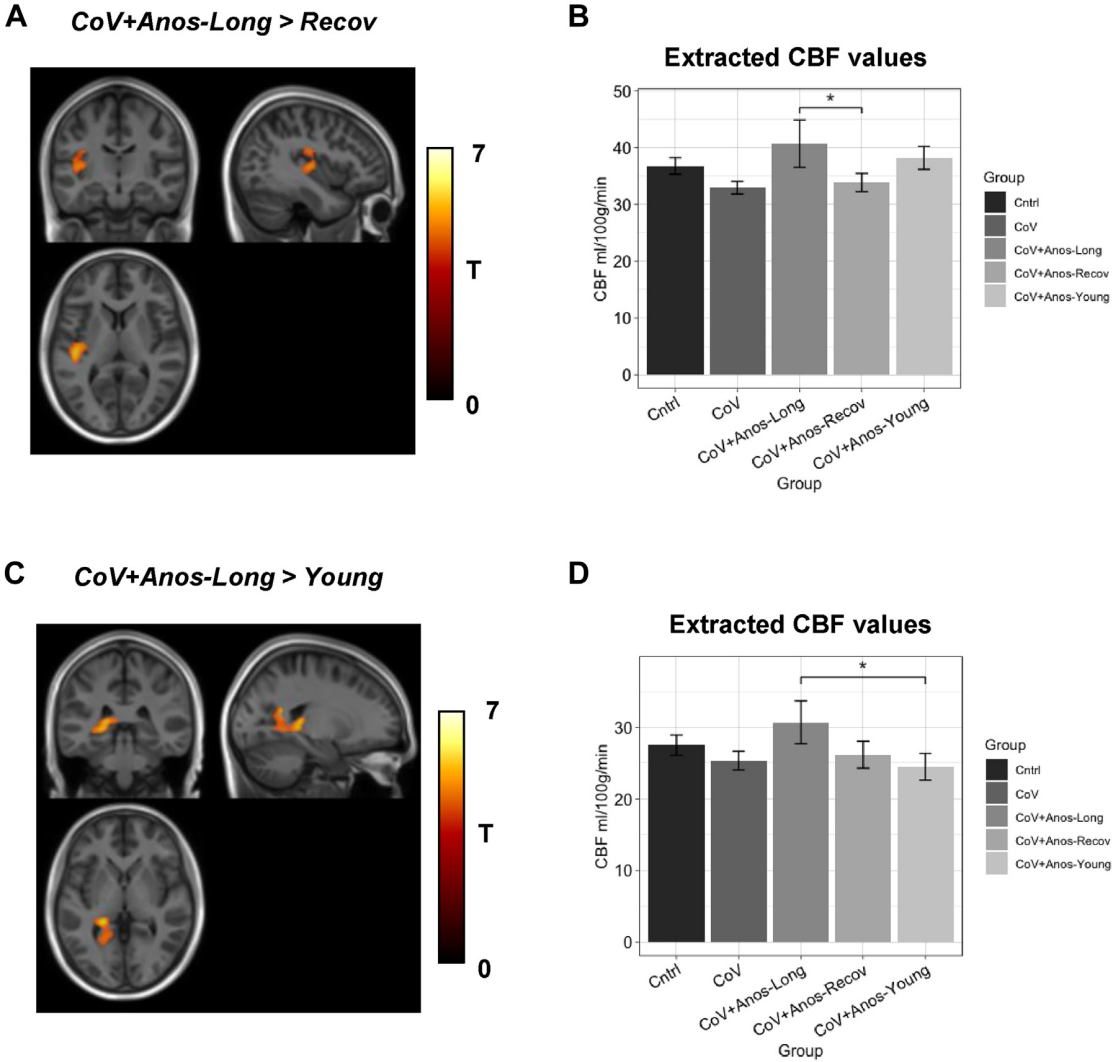
**A) In hot colours, significant clusters overlaid on the MNI template showing increased CBF for a region of the left insula in the *CoV+Anos-Long* group compared to the *CoV+Anos-Recov* group. B) Mean CBF extracted from the left insula cluster for each group. C) Significant clusters overlaid onto an MNI template showing increased CBF for subcortical and cortical areas including the left Thalamus, posterior hippocampus and ventral posterior cingulate in the *CoV+Anos-Long* group compared to the *CoV+Anos-Young* group. D) Mean CBF extracted from the cluster in (C) for each group.** ∗ denotes the significant group comparisons for whole brain analysis, statistics was not performed on extracted values. Both Statistical Parametric Maps were obtained using a cluster significance threshold set to *p* < 0.05. CBF – Cerebral blood flow in units ml/100g/brain tissue.

The *CoV+Anos-Long > CoV+Anos-Recov* contrast provided a large significant cluster (459 voxels, *p* < 0.01, *T* = 4.99) which spanned a region of the left insula cortex (MNI co-ords: −44, −22, 4) (see Fig. 4A). In addition, the *CoV+Anos-Long > CoV+Anos-Young* contrast provided a large significant cluster (952 voxels, *p* < 0.001, *T* = 5.26), which spanned several subcortical and cortical areas including the left thalamus, posterior hippocampus and ventral posterior cingulate (MNI co-ords: −22, −38, 0) (see Fig. 4C). No additional group CBF differences were observed.

### Whole brain structural analysis

**Fig. 5:**
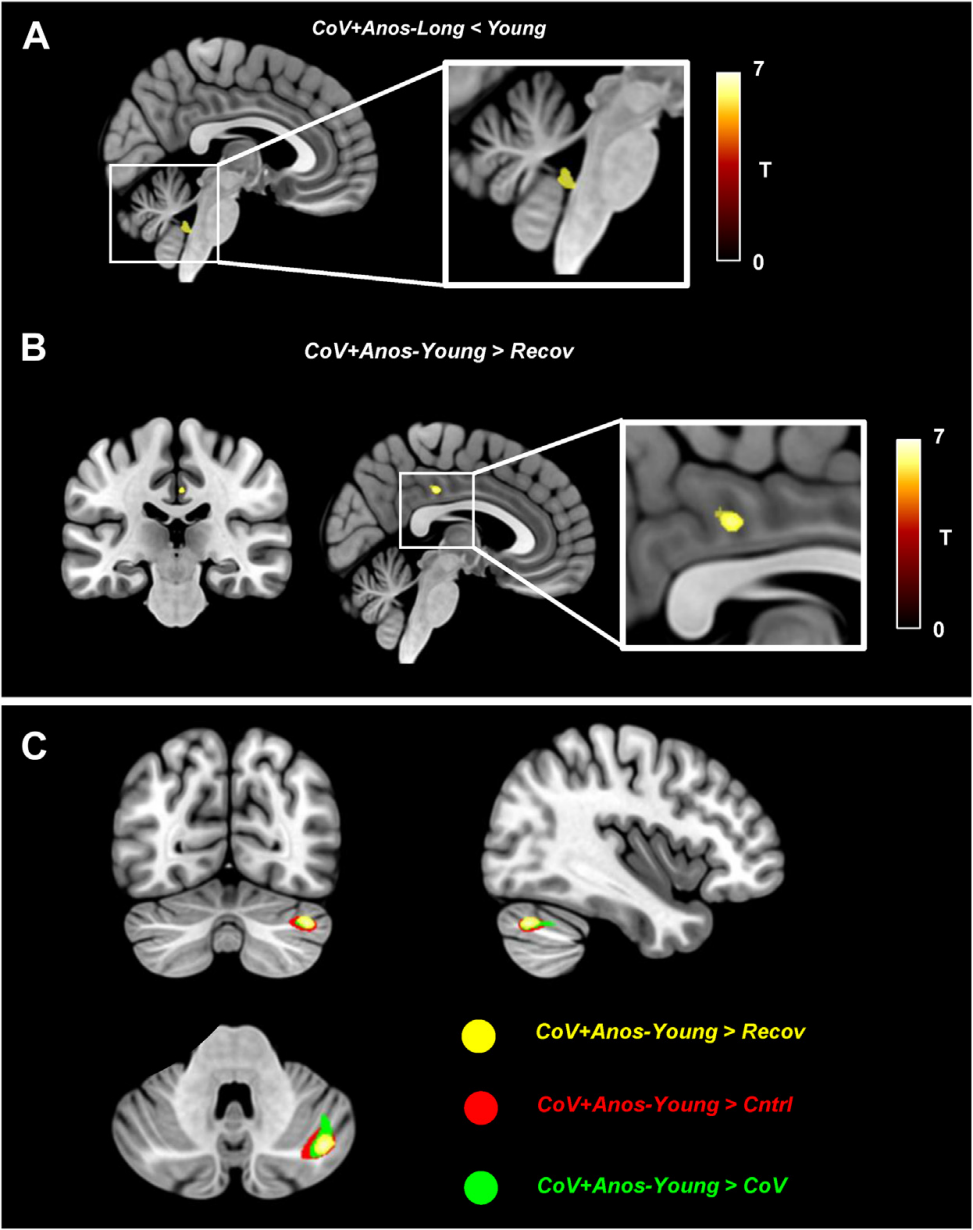
**A) In hot colours, a significant cluster overlaid on the MNI template showing reduced GM density for a region of the brainstem/cerebellum in the *CoV+Anos-Long* group compared to the *CoV+Anos-Young* group. B) A significant cluster overlaid onto an MNI template showing increased GM density in a region of the right ventral posterior cingulate for *CoV+Anos-Young* group compared to the *CoV+Anos-Recov* group. C) Significant clusters in the cerebellum showing increased GM density in this region for the *CoV+Anos-Young* group compared to *Cov+Anos-Recov* (yellow), *Cntrl* (red) and *Cov+* (green) groups.** All Statistical Parametric Maps were obtained using a cluster FWE significance threshold set to *p* < 0.05.

All structural morphometry results are presented in Table 3. From whole brain voxel based analysis of structural T1-weighted data we observed differences in GM density across groups. Firstly, *CoV+Anos-Long* individuals showed reduced GM density compared to the *CoV+Anos-Young* group in a region of the posterior brainstem which spanned to the right IX region of the cerebellum (Fig. 5C). No further GM differences for the *Cov+Anos-Long* group were identified between any other group.

**Table 3:** Voxel based morphometry results.

Contrast	Region	MNI co-ordinates			Cluster size (voxels)	*p* value
		x	y	z		
CoV+Anos-Long < CoV+Anos-Young	Right IX/brainstem	5	−44	−37	180	<0.001
CoV+Anos-Young < CoV+Anos-Recov	Right vPCC	4	−28	36	68	0.006
CoV+Anos-Young < CoV+Anos-Recov	Right Crus I	40	−68	−32	249	<0.001
CoV+Anos-Young < Cntrl	Right Crus I	40	−68	−33	740	<0.001
CoV+Anos-Young < CoV	Right Crus I	40	−68	−32	407	<0.001

vPCC: ventral posterior cingulate cortex, Cntrl: Healthy controls, CoV: Previous COVID-19 infection, CoV+Anos-Long: Long COVID-19 Anosmia, CoV+Anos-Recov: Recovered COVID-19 Anosmia (age matched to CoV+Anos-Long) and CoV+Anos-Young: Young and Recovered COVID-19 Anosmia group.

The *CoV+Anos-Young* group displayed an increased GM density compared to *CoV+Anos-Recov* individuals in a region of cortical GM, i.e. the right ventral posterior cingulate cortex (vPCC) (Fig. 5B).

In addition, the *CoV+Anos-Young* group showed a statistically greater GM density in a region of the cerebellum, the right Crus I, which differed from *CoV+Anos-Recov, Cntrl* and *Cov* groups. These significant clusters show a spatial overlap as seen in Fig. 5A.

## Discussion

This work describes an explorative observational study, which has utilised multi-modal MR imaging techniques to assess how COVID-19 infection and related long-COVID anosmia impacts on resting state FC, cerebral blood perfusion and GM density. CBF data showed changes amongst the three COVID-19 Anosmia groups; CBF within the left insula was increased in *long COVID Anosmia* individuals vs the age-matched *Recovered* group and a CBF increase within the left thalamus, posterior cingulate cortex in *Long* *COVID Anosmia* individuals compared to the *Young Recovered* group. Significant increases in FC between the *Long* *COVID Anosmia* group and our *healthy control* individuals were found from seed-based analysis. One of these FC increases described elevated FC between the R-OFC seed and an area of the right visual association cortex and fusiform gyrus, which was also significantly elevated compared to the *COVID-19* group too. In addition, reduced FC between the L-OFC and a region of the left dorsal ACC was observed in the *Long COVID Anosmia* group compared with *healthy control* individuals. The ACC forms a key part of the executive network and our dual regression analysis of the executive network found reduced FC in this prefrontal region for *Long COVID Anosmia* vs control group comparisons.

The OFC, like the other seeds in this analysis, forms one of the bilateral nodes within a resting state olfactory network.^12^ The OFC is described as a secondary olfactory region (the olfactory bulbs being the primary regions) and functionally a sensory convergence centre, not only receiving input from olfactory but visual and taste based stimulus too.^22^ In addition, the OFC is responsible for processing information about the identity as well as the reward value associated with odours.^22^ Interestingly the L-OFC seed region showed a different pattern of FC differences between *Long* *COVID Anosmia* individuals and *healthy controls*. Reduced FC between the L-OFC and a region of the left dorsal ACC was observed in the *Long* *COVID Anosmia* group. Anatomical data from primates report direct connections via a monosynaptic pathway between this OFC region and the ACC.^23^ Moreover, the ACC is thought to control or modulate an individual's attention for and towards olfactory stimuli.^23^ These data support the hypothesis that persistent hyposmia/anosmia may reduce attentional processing towards olfactory stimuli and perhaps the sustained lack of olfactory attention or sensing underlies the reduced FC observed between these regions in our data. Plotting the FC values between the L-OFC and the left dorsal ACC highlighted that the other COVID-19 positive groups had lower, but not significantly lower, values, suggestive of a more generalised altered FC profile between these regions. Importantly, these reductions in FC were not associated with any local structural changes or modifications suggesting that these could be reversed by approaches such as olfactory training,^24^ an approach which has been encouraged and successfully employed for COVID-19 related smell loss.^25^^,^^26^

FC changes in the both the left and right OFC show further support, more generally, for the OFC and its involvement in COVID-19 infection, which was one of the main anatomical regions reported from the UK Biobank COVID-19 neuroimaging study.^13^ Further interrogation of our data showed that the measured connectivity strength (Z values) between the R-OFC and the visual association cortex in control individuals was low (less than 0.1), suggesting that these regions are not strongly connected under healthy conditions. Visualisation of our data shows that there is a clear significant increase in FC within the *Long* *COVID Anosmia group*, suggestive of an altered connectivity profile. Furthermore, these visual processing regions are not commonly reported in relation to olfactory processing networks.^8^^,^^12^ *Kollndorfer* et al., also found connectivity profiles beyond those of the olfactory network in patients with smell loss,^27^ which dissipated following olfactory training and upon regaining olfactory processing abilities. It has also been suggested that a lack of olfactory processing may stimulate functional connections between olfactory nodes and other sensory regions or more generally describe compensatory mechanisms, and has been previously described in those who have acquired anosmia (rather than from birth).^10^ Using dynamic connectivity measures *Iravani* and colleagues studied FC between one of the olfactory nodes, the piriform cortex, and found that anosmia patients, not caused by COVID-19, was associated with increased FC to other multi-sensory processing regions, namely visual.^10^ Although we did not observe any functional differences from the piriform cortex seed analysis our data supports this finding with an increase in FC between an olfactory node (right OFC) and a visual region (right visual association cortex). Sensory deprivation, such as blindness, displays a similar pattern, described by some as a compensatory mechanism.^28^ It is unknown to what degree this observed connectivity profile impacts on behaviour or contributes to functional changes. Equally important, no differences in FC profiles were observed between healthy controls and individuals who recovered from olfactory deficits, young and older, suggesting that this hyper-connectivity profile may subside following symptom recovery.

In addition, our analysis showed increases in FC between the left anterior insula seed and the left Crus I region of the cerebellum, an increase which upon interrogation looked to be a connection not found within the healthy controls. The Crus I region, however, has been identified as part of the olfactory network and is recruited during active olfactory processing, in particular when smelling unpleasant odours,^29^ which may be why this region was not seen within our resting state analysis in healthy controls. Similar to the OFC, the insula has also been recently highlighted from the UK Biobank study as a region that displays longitudinal structural reductions from COVID-19^13^ as well as the ACC. The ACC has been observed in olfactory processing,^30^ with the dorsal portion having an involvement in a number of cognitive functions and is more generally associated as a neurobiological substrate for mental illness.^31^ Interestingly, network level analysis showed that the executive control network identified increased FC between the *Long* *COVID Anosmia* and *Control* individuals in the left and the right dorsal ACC. The significant cluster found sits within the central node of the executive network and this work should encourage analysis in future studies to asses higher level cognitive impact associated with long-COVID.^32^ Some of the main symptoms associated with long COVID, in addition to anosmia, relate to cognitive impairment^33^ such as brain fog, which impacts on attention, executive functioning and decision making. The degree to which these patients may have experienced these non-olfactory symptoms was not explored in this work, as at the time of scanning long COVID was just emerging and clinical tests for assessing non-sensory impairment were not put in place in our ethics protocol. However, these results may suggest that we should not underestimate a potential relationship or overlap of more cognitive long COVID symptoms and anosmia. Data collection for all patients in this study was taken some time after COVID-19 infection and so it is unknown how these FC changes manifest and whether they were present at the time of infection or whether they developed in the post-acute/chronic period.

The significant FC alteration of the cerebellum is consistent with results showing that the cerebellum is involved in olfactory networks. Crus-I activates during olfaction^29^ and is part of an olfacto-cerebellar pathway with a prominent role in odour identification and detection, functionally connecting each nostril primarily to the contralateral cerebellum.^34^ The emerging FC of cerebellum with the insula is especially interesting, since the cerebellum plays a primary role in error-based learning that extends over multiple sensory and cognitive domains^35^ and is involved within the main executive networks (Default mode, Salience and Executive network).^36^ Thus, alterations in the olfactory networks eventually involve connected nodes also in the hindbrain, extending from OFC and ACC to the cerebellum.

ASL was used to interrogate changes in regional GM CBF. Global GM perfusion did not significantly differ across groups and was used as a covariate of no interest for the analysis along with age and sex (which were used for all other analysis too). The lack of global GM perfusion differences permits the interpretation that observed differences in regional CBF are the result of local changes in neuronal activity/metabolism, due to the generally accepted coupling between blood flow and metabolism.^37^ Our data shows group differences between *Anosmia* groups, with the *Long* *COVID Anosmia* group displaying greater regional CBF in the left posterior insula cortex compared to the age matched *COVID Anosmia Recovered* group. In addition, greater regional CBF was found for a cluster that overlapped the boundaries of the left thalamus, posterior hippocampus and ventral cingulate in the *Long* *COVID Anosmia* vs the *COVID Anosmia Young* group. These regions are involved in relaying sensory information to the sensory cortex, memory recall/processing^38^ and the ventral cingulate forms part of the default mode network,^39^ respectively. All of these regions are supplied by the posterior cerebral artery.^40^ What's driving these perfusion differences is hard to pinpoint from our imaging data alone. However, from an anatomical perspective, the posterior artery which supplies some of these regions is smaller in diameter compared to the other cerebral feeding arteries. A neurological finding which has been observed in severe COVID-19 patients is the presence of micro-haemorrhages and neurovascular effects.^41^ To what degree COVID-19 impacts neurovascular integrity in mild to moderate cases and indeed in long COVID is largely unknown. However, a speculative inference of the CBF findings shown here may be due to subtle vascular effects that are only noticeable in the smaller diameter arteries supplying the brain. We did not observe CBF differences between the *healthy control* group and any of the COVID-19 groups. This is somewhat reassuring that baseline resting perfusion remains unimpacted by acute COVID-19 infection and also those with long COVID symptoms.

Structural image analysis, using voxel-based morphometry, showed differences in GM density across the groups, however, none of the structural differences identified related to any of the structures or regions identified in the FC analysis. We observed GM reductions for a region of the cerebellum, the Right IX, in the *Long COVID Anosmia* group when compared with the *Young Recovered Anosmia group*. In addition, this *Young Recovered* group had greater GM density compared to the *Recovered Anosmia, healthy controls and COVID-19* groups for a region of the cerebellum, the right Crus I. Regional GM density differences for this region were seen across both positive and negative antibody groups which would suggest that this effect is not a consequence of having prior COVID-19 infection. The *Young Recovered Anosmia* group were, as the group name suggests, significantly younger in age compared to all the other groups and despite adding age as a covariate in our analysis we can't confidently say that this observed result is not a GM density difference driven by age. This exploratory VBM analysis focused on global GM, but perhaps given these findings in the cerebellum it would be of value to use a cerebellum based spatial normalisation pipeline, such as the spatially unbiased infratentorial template (SUIT) toolbox.^42^ Interestingly, there was also a cortical GM density difference in the cerebrum seen between the two *Anosmia Recovered* groups, with the younger of the two groups displaying a greater density in a small cluster situated within the right ventral posterior cingulate cortex. Had this region been identified between the *Long* *COVID* and younger group as well it may support an effect of age, which is observed even when accounting for it. The lack of differing characteristics between these two recovered groups impedes the interpretation. Despite the relatively small sample size used in this study, previous work looking at reproducibility of VBM did not suggest a small sample size impacted on false positive rate.^43^

Our study has several limitations. Our dataset strongly relies on neuroimaging data and does not contain additional behavioural measures which may support and strengthen the results presented. In addition, given the data were collected during COVID restrictions we had limited ethical approval to collect information on comorbidities or anthropometry during this study. Another limitation to this observational study is the sample size that was used, thus categorising this study as exploratory in nature. In addition, all our COVID-19 anosmia suffering groups are imbalanced with regards to sex, with all groups consisting of a higher ratio of female individuals compared to males. We adjusted for the potential impact of this unbalance in our statistical models using appropriate covariates. Regardless of this unequal ratio, data has shown that the prevalence of anosmia and olfactory dysfunction from COVID-19 is far higher in females compared to males,^3^ a finding which was also confirmed by our previous community based cohort work and from where these individuals in this imaging study were recruited from.^1^^,^^14^ We argue that given the unbalanced effect seen in both *Makarondis* et al. and across larger cohort studies that our sample of individuals studied is representative, thus permitting confidence in our interpretations.^1^^,^^14^ In addition to sex, age has also been shown to have an impact on anosmia recovery, with higher rates of olfactory recovery following COVID-19 occurring in younger people compared to older individuals.^14^ As such our *Long* *COVID* *Anosmia* group were of an older age which is in accordance with the associations seen across the community and in larger populations. We have highlighted that this observational study is also a cross-sectional design, and we are limited in terms of not having baseline (pre-COVID) imaging data for which each individual can be used as their own control for comparisons. This is a limitation for many studies looking at the impact of COVID-19 and for our work we have recruited a healthy control group to aid our inferences and analysis. In addition, our data does not contain follow up and/or serial measurements for this group. Access to follow up data in this group would be useful to see if these changes in brain function returned to normal or if any behavioural changes developed in relation to the specific connectivity and perfusion changes identified here. Follow up data and a larger number of individuals may hopefully allow for neural substrates or connectivity signatures to be identified, offering an ability to predict symptom outcomes and is a recommendation for future studies looking at long COVID-19 impact. One final limitation is that the UPSIT tests were only assessed in those individuals recruited from the *Makaronidis* et al. cohort.^14^ Due to restrictions in place in the UK at the time of the scanning we were unable to collect this data in healthy control individuals, which has limited the use of these UPSIT scores for additional correlational analyses.

Our work describes a novel approach to examine the long-term impact of smell loss from COVID-19, which is beyond that of case study reports and adds considerably to the current literature. Moreover, our work is the first to combine multi-modal MRI methods for the investigation of long-term symptoms and impact of COVID-19 on the brain. We have presented data on brain structure, functional connectivity as well as cerebral perfusion, all of which add new knowledge to the current understanding. To conclude, we have found differences in functional connectivity from olfactory nodes that exist between COVID-19 naïve individuals and those who still suffer from olfactory dysfunction following COVID-19 infection. Our data highlight some differences in brain perfusion in areas supplied by the posterior cerebral artery and a greater in-depth exploration of these areas and vascular integrity is needed.

## Contributors

JW processed and analysed data, interpreted results and wrote the manuscript. JM conceived and designed the study, recruited patients, collected data and wrote the manuscript. FP and BK processed and analysed data and edited the manuscript. MY collected imaging data and edited the manuscript. CM was involved in the data collection. GC and XG helped setting up the scan protocol and editing the manuscript. CT gave statistical support to the study and edited the manuscript. OC and ED'A contributed to early discussions about the study, helped interpreting the data and edited the manuscript. CGWK and RLB conceived the study, designed experiments, interpreted results, wrote the manuscript and verified the data. All authors are responsible for the decision to submit the manuscript and all authors had access to the data and have read and approved the final version of the manuscript.

## Data sharing statement

De-identified participant data will be made available after publication by application to the corresponding author and after assenting to a data access agreement.

## Declaration of interests

XG is a founder, shareholder and CEO of Gold Standard Phantoms, a company providing calibration and test objects for MRI. ED’A received funding from the H2020 Research and Innovation Action Grants Human Brain Project 785907 and 945539 (SGA2 and SGA3), and from the MNL Project “Local Neuronal Microcircuits” of the Centro Fermi (Rome, Italy). CGWK received funding from the H2020 Research and Innovation Action Grants Human Brain Project 945539 (SGA3), from the MS Society (#77), from Wings for Life (#169111), BRC (#BRC704/CAP/CGW), MRC (#MR/S026088/1), Ataxia UK. CGWK is a shareholder in Queen Square Analytics Ltd. RLB reports receiving consulting fees from Pfizer, Eli-Lilly, Gila Therapeutics Inc., and ViiV Healthcare and consulting fees, lecture fees from Novo Nordisk and participating in clinical trials for Novo Nordisk. RLB receives National Institute for Health and Care Research Professorship funding RP-2015-06-005 and JW is funded under this funding too. JM receives UCL/UCLH National Institute for Health and Care Research BRC funding.

## Appendix A Supplementary data

COBIDASreport_companion_file
